# Correspondence

**Published:** 1865-04

**Authors:** 


### CORRESPONDENCE.

BELLEVILLE, C. W., March 8th, 1865.

To the Editor of the Buffalo Medical and Surgical Journal:

Sir:—In the *Canada Medical Journal* for March, I find an interesting case recorded, of "Resection of the Ankle-joint," by Dr. John G. Johnson, taken from the *Buffalo Medical Journal.* Perhaps your readers, including your correspondent Dr. Johnson, will be interested to read the accompanying article, copied from the *London Lancet*, page 256, vol. 2, 1864.

I may add that this case is referred to in *Braithwaite* for July, 1863.

Truly yours, W. CANNIFF.

### "RESECTION OF THE ANKLE-JOINT."

"To the Editor of The Lancet:

"Sir:—I had the honor to report in the *British American Journal*, in the June number of 1862, a case of "Resection of the Ankle-joint." The case was recorded four months after the operation. I then stated that I should at the expiration of a year furnish the profession with the final result. The Journal in which the case was published has ceased to exist. I therefore take the liberty of requesting a space in *The Lancet* to fulfill my promise. Indeed I venture to think the case of sufficient importance to warrant its publication in your widely circulated Journal.

There are two or three points to which I purpose particularly to refer, and which will become apparent as I proceed. These are points regarding which there is at the present time perhaps no settled opinion. The operation was performed in the way recommended by Henry Hancock, Esq., Surgeon to the Charing-Cross Hospital. Last winter, Mr. Hancock, in urging the importance of this operation, referred to the success of the case under consideration, and I think from it it will be seen that conservative surgery deserved to be more fully tested. The young man operated upon was 21 years of age, with a constitution in most respects good. The disease of the bones which led to the operation was, I think, chiefly, if not altogether, due to local causes. Having made the single incision through the integument, as recommended, and dissected up the flap, the following pieces of bone were successively removed: First, the astragalus, one-half of which was in a state of necrosis, and the other portion in a disorganized condition. When the external malleolus, after which the tibia was turned out, and a little more than half an inch sawn off; but the condition of the bone above was such that it was deemed necessary to remove more; consequently, the incision through the soft parts having been extended, and the parts dissected from the bone, an inch and a half more of both tibia and fibula were removed. At this time it was recommended by the surgeon assisting me, to amputate; but as I had a strong faith in nature's ability to heal, and as the patient had caused me to promise that if there was but a slight possibility of saving the foot, to give him the benefit of it, I determined to make the trial. The upper surface of the os-calcis was thereafter also removed to the extent of half an inch by the gouge. The operation being completed, the limb was placed in the fracture-box. By careful measurement of the bones excised, it was found that fully three inches in length had been removed. The space, however, between the bones of the foot and those of the leg were perceptibly diminished by contraction of the muscles of the leg. I confess my anxiety was great to see the result, for I was not aware of any precedent for so extensive a removal of bone. But no artery of any size had been divided in the operation; the diseased bone had been completely removed, and I trusted the powers of nature would prove adequate to the task of repair, although so extensive. A portion of the flap, in which was a cicatrix, that had resulted from previous sloughing, subsequently perished, leaving a large opening, through which could be seen the end of the tibia. But, notwithstanding this increased work of repair, healing of the whole rapidly progressed. There was no great discharge at any time, no inflammation, no waste of separative material. Water dressing alone was used. I mention these facts, because had there been irritation, had there been much discharge, the result might not have been so favorable.

At the end of nine weeks the limb presented the following appearance: A healthy looking and limited cicatrix, marking the incision through the integument. A very small opening internally, where had been the most sloughing. The foot reduced to almost its natural size, and in a natural position. There is by admeasurement not more than an inch and a half of shortening. The bones of the foot have not yet joined with those of the leg. The foot can be moved passively in any direction, yet there is a comfortable degree of firmness, which has been constantly increasing. The patient can move the foot and toes in a natural manner. In a word, there is a prospect of an excellent joint.

Within four months after the operation he could rest the foot upon the ground; within six months he could walk by the aid of a cane, and at the expiration of a year he could run upon it. I saw him on one occasion mount a flight of stairs, three steps at a leap. I have recently seen the patient, and found by measurement there is just two inches of shortening. A boot is worn, with a sole thickened about three-quarters of an inch. The ankle is supported on either side by steel springs, and he walks with a very slight limp.

Now in this case we have a very striking exemplification of the resources of nature. Not only is there an extraordinary restoration of bone, but there has also been the formation of a new joint. In order to prevent an ossific union between the bones of the foot and those of the leg, the patient was instructed at an early date to exercise the muscles of the leg, so as to cause active motion. The result now is a very perfect joint. Not a few medical friends, with whom I have conversed about it, cannot credit the fact that there is any motion, being led away with the belief that after excision of a joint there is necessarily a stiff joint. I have reminded such that a very common cause of false joint after fracture is continued motion at the seat of fracture; and if so unfortunate a result follows so simple a cause in connection with a fracture badly treated, why can we not secure the same result after excision of a joint, by which the natural motion and use of the limb will be retained? This, as I have before said, can be secured by causing the patient to use the muscles, so as to keep up motion. One can only speculate with regard to the provisions of this new joint, and it would be interesting and instructive to examine it; but in this case I do not think a chance will offer as long as the patient lives.

This operation, I believe, was never before performed on this side the Atlantic. During the past summer, while sojourning a couple of months at Washington, I learned that the operation was never practiced in the service; and although very many cases presented themselves where it might be performed, amputation was always resorted to instead.

I remain, Sir, yours, etc.,

WM. CANNIFF, M. D., M. R. C. S. Eng.

Formerly Act. Ass't Surg. to her Majesty's Forces; late Professor of Surgery, University Vict. College, Toronto.

*Belleville, Canada West*, 1864."

